# NCKAP1 is a Prognostic Biomarker for Inhibition of Cell Growth in Clear Cell Renal Cell Carcinoma

**DOI:** 10.3389/fgene.2022.764957

**Published:** 2022-07-26

**Authors:** Jiasheng Chen, Jianzhang Ge, Wancong Zhang, Xuqi Xie, Xiaoping Zhong, Shijie Tang

**Affiliations:** ^1^ Department of Burns and Plastic Surgery, The Second Affiliated Hospital of Shantou University Medical College, Shantou, China; ^2^ Department of Urology, Changsha Central Hospital, Changsha, China

**Keywords:** clear cell renal cell carcinoma, Nckap1, biomarkers, prognosis, progression

## Abstract

**Background:** Clear cell renal cell carcinoma (ccRCC) is the most frequent type of kidney cancer. Nck-associated protein 1 (NCKAP1) is associated with poor prognosis and tumor progression in several cancer types, but the function and prognostic value of NCKAP1 in ccRCC remain poorly understood.

**Methods:** Using the Ualcan database, we evaluated the correlation between NCKAP1 expression and clinical features of ccRCC. These data were validated by immunohistochemical staining for NCKAP1 in a cohort of ccRCC patients. We assessed the prognostic value of NCKAP1 using GEPIA2 survival analysis. NCKAP1 function was characterized *in vitro* and *in vivo* using NCKAP1-overexpression ACHN cell lines. The LinkedOmics and GSCALite databases were used to investigate identify potential NCKAP1-targeted medicines that may play a role in the treatment of ccRCC. The impact of NCKAP1 expression on immune infiltration was also evaluated.

**Results:** NCKAP1 was significantly downregulated in ccRCC and correlated with advanced clinicopathological features and poor prognosis. Overexpression of NCKAP1 in ACHN cells reduced proliferation, invasion and migration capacity *in vitro* and inhibited tumor growth *in vivo*. According to the LinkedOmics, GSCALite and TIMER databases, NCKAP1 and related genes function primarily in ribosomal signaling, oxidative phosphorylation, TGF-β, and EMT-related signaling pathways. NCKAP1 was also shown to positively correlate with immune cell types, biomarkers, and immune checkpoints in ccRCCs.

**Conclusions:** NCKAP1 may play a vital tumor-suppressive role in ccRCC and is potentially a useful prognostic biomarker.

## Introduction

Renal cell carcinoma (RCC) is a malignant tumor of the urinary system and accounts for about 3% of cancers worldwide (Kotecha et al., 2019). Based on the World Health Organization (WHO) classification system, RCC in adults is classified into four types: clear cell, papillary, pigmented, and collecting duct type, of which clear cell RCC (ccRCC) is the most common type worldwide (Patel et al., 2012). Over the past few decades, the incidence of RCC has increased by 2% per year, due in part to the difficulty of early diagnosis, and approximately 25% of patients present with metastatic disease (Perazella et al., 2018; Capitanio et al., 2019). There is a tremendous unmet need for developing novel diagnostic biomarkers and therapeutic targets that have the potential to improve the prognosis of RCC patients.

NCK-associated protein 1 (NCKAP1) is a protein found in sporadic Alzheimer’s disease (AD) as part of the WAVE complex along with ABI1-2, BRK1, CYFIP1-2, and WASF1-2 proteins (Suzuki et al., 2000; Innocenti et al., 2004). NCKAP1 regulates various intracellular processes such as apoptosis, migration, and invasion, and plays an essential role in disease pathogenesis (Whitelaw et al., 2020). NCKAP1 expression is highly tissue-specific, and its expression has been found in colon, breast, and lung cancers (Teng et al., 2016; Xiong et al., 2019a; Rai et al., 2020; Zhang et al., 2020). On the other hand, we previously showed that downregulation of NCKAP1 in liver cancer patients is associated with poor prognosis (Zhong et al., 2019). These data suggest that NCKAP1 may have tumor-promoting or suppressive effects on certain types of cancer. However, the clinicopathological characteristics and function of NCKAP1 in ccRCC have not yet been confirmed.

In this study, we aimed to determine the function of NCKAP1 in ccRCC using bioinformatics analysis portal tools and immunohistochemical validation, to examine the relationship between NCKAP1 expression and clinicopathological features of ccRCC, and to determine the *in vitro* and *in vivo* NCKAP1 expression was measured to characterize the clinicopathological features of ccRCC. We also determined the *in vitro* and *in vivo* role of NCKAP1 in a related cell line (ACHN). In addition, the predicted functions of NCKAP1 and tumor immune infiltrating cells were discussed.

## Materials and Methods

### Patients and Samples

Shanghai Xinchao Biotechnology (Shanghai, China) was the commercial source of renal cell carcinoma tissue microarray (TMA). The staging of tumors was done employing the 2010 revised TNM system and the TMA consisted of stages I-II disease (*n* = 52) and stage III-IV (*n* = 23) disease. The WHO criteria were utilized to specify the histological grades of tumors as mentioned, low grade (Grade I and II; *n* = 55) and high grade (Grade III and IV; *n* = 20).

### Bioinformatics Analysis

NCKAP1 mRNA, protein expression and the associated clinical features were examined in ccRCC using the UALCAN database (Chandrashekar et al., 2017) (http://ualcan.path.uab.edu/). Gene Expression Profiling Interactive Analysis (GEPIA) (Tang et al., 2017) (http://gepia.cancer-pku.cn/index.html) was used to analyze the survival information between NCKAP1 and ccRCC. The LinkedOmics database (Vasaikar et al., 2018) (www.linkedomics.org) was used to analyze the genes that significantly correlated with NCKAP1, GO enrichment, KEGG pathways, kinase targets and miRNA targets in ccRCC. GSCALite (www.bioinfo.life.hust.edu.cn/web/GSCALite/) was used to analyze and visualize the gene sets correction with pathway activity in our study with TCGA ccRCC sample. TIMER (Li et al., 2017) (www.cistrome.shinyapps.io/timer/) was employed to probe components of tumor immune cell characteristics. Immune Checkpoints, TMB, MSI R package were implemented by R foundation for statistical computing (2020) version 4.0.3 and software packages ggplot2 and pheatmap (Thorsson et al., 2019).

### Immunohistochemical Analysis

Immunohistochemical (IHC) staining of NCKAP1,Ki-67 and E-cad were carried out as previously reported (Zhong et al., 2019). The TMA and xenograft tumor sections were incubated with the NCKAP1, Ki-67, E-cad antibody (1:100; Proteintech, China). Stained sections were then independently assessed by two pathologists.

### Cell Culture and Transfections

The American Type Culture Collection (ATCC, Manassas, VA, United States) was the source of human RCC cell lines ACHN, 786-O, and 769-P. The OS-RC-2 cell line was received as a gift from the Cancer Research Center of Shantou University Medical College (Shantou, China). Culture of the cell lines was done in Roswell Park Memorial Institute medium (RPMI-1640, Gibco, Gaithersburg, MD) supplemented with 10% fetal bovine serum (FBS; Gibco, United States) and maintained in an atmosphere of 5% CO_2_ atmosphere at 37°C.

Hanbio Biotechnology Co., Ltd. (Shanghai, China) was the commercial source of alentiviral NCKAP1 overexpression vector and an empty vector. ACHN cells were transfected using Lipofectamine 2000 and Opti-MEMI (Gibco, United States) in accordance with the prescribed protocol of the manufacturer.

### qRT-PCR Analysis

Trizol reagent (Tiangen Biotech, China) was employed to extract the total RNA and cDNA synthesis performed using a Revert Aid First Strand cDNA Synthesis Kit (Thermo Scientific, United States). qRT-PCR analysis for the expression of NCKAP1 was performed in triplicate using SYBR Green I (Tiangen Biotech, China) in accordance with the prescribed protocol of the manufacturer. The internal control was GAPDH. The primer sequences for NCKAP1 and GAPDH were as follows:

NCKAP1
   5′-TCCTAAATACTGACGCTACAGCA-3′(forward)
   5′-GCCTCCTTGCATTCTCTTATGTC-3′(reverse)
GAPDH
   5′-GTCTCCTCTGACTTCAACAGCG-3′(forward)
   5 ′-ACCACCCTGTTGCTGTAGCCAA-3′(reverse)

### Western Blotting

Proteins were isolated using a whole-cell lysis assay (Beyotime Biotechnology, Jiangsu, China). Following the resolution of the protein samples by sodium dodecyl sulfate-polyacrylamide gel electrophoresis (SDS-PAGE) transfer to polyvinylidene difluoride (PVDF) membranes was done for western blotting. Membranes were blocked with skimmed milk followed by NCKAP1 primary antibody (1:1,000; Proteintech, China) overnight incubation at 4°C. Subsequent secondary antibody (1:5,000; Abcam) was done at room temperature following membrane washing. Blots were developed using a chemiluminescence detection kit. GAPDH (1:1,000; Proteintech, China) was used as the loading control.

### Cell Viability Assay

A Cell Counting Kit-8 (Beyotime Biotechnology, Jiangsu, China) was employed to quantify the cell viability in accordance with the prescribed approach. Briefly, 96 well plates were used to seed 2 × 10^3^ cells followed by 5 days of culture under normal conditions. At the end of each period (1, 2, 3, 4 or 5 days), incubation of the plates was done at 37°C for 2 hours following the addition of 10 μL of CCK-8 to each well. Absorbance values measured at 540 nm to quantify the cell viability. Triplicate assays were conducted and repeated three times.

### Colony Formation Assay

6 well plates were employed to seed 1 × 10^3^ cells followed by incubation at 37°C. After 2 weeks of culture, 4% paraformaldehyde was utilized to fix colonies and subsequent crystal violet (0.5%) staining at room temperature. Enumeration of the colonies was done utilizing digital images of the well obtained from each of the three replicate wells.

### Wound Healing Assay

This was done to evaluate the ability of the cells to migrate. Briefly, 24 well plates were utilized to seed 5 × 10^5^ cells and allowed to adhere overnight. Cells grew to confluence and then artificial wounds were introduced using to mark a line down the center of the cell layer. Cells were then cultured in serum-free medium. The wounded areas were imaged right away (0 h) and at 24 h after the wound was introduced using an inverted microscope (Olympus Corp). Triplicate experiments were done.

### Transwell Assay

In order to quantify cell invasion this assay involved the coating of Transwellinserts (8 μm pore size, Corning, NY, United States) with matrigel (BD Biosciences, NJ, United States) and 5 × 10^4^ cells were added into the upper compartment. RPMI-1640 containing 20% FBS was then added to the lower chamber of the transwell and the cells followed by a 24-h incubation. Post-migration of cells to the lower chamber from the upper chamber, the membranes were then stained and the migrated cells were enumerated. The number of cells was scored from five randomly selected fields of view on the lower membrane. The assay was performed in triplicate.

### Animal Experiments

Male BALB/c athymic nude mice (4–6 weeks old) were purchased from Hunan SJA Laboratory Animal Co., Ltd. 3.0 × 10^6^ cells transfected with ACHN-NCKAP1 or ACHN-vector were injected subcutaneously into mice to set up the ccRCC xenograft model. When tumors were palpable, their sizes were measured every 3 days for 14 days. 2 weeks post-monitored, the sacrifice of both groups of animals was done and tumors were isolated for growth and IHC analyses. Tumor dimensions were gauged employing calipers and the volumes were calculated utilizing the expression V = (shorter diameter^2^ × longer diameter)/2. These experiments were approved by the Ethics Committee of Shantou University Medical College.

### Statistical Analysis

The outcomes of the survival curve, GEPIA databases are represented by the HR and *p* or the COX *P*-values of a log-rank test. Assessment of the correlation of gene expression was done using the LinkedOmics, GSCALite and TIMER databases and compared with Pearson Correlation analysis. The Pearson χ^2^ test was utilized for quantifying the correlation between the expression of NCKAP1 and the patient clinic-pathological parameters. Other data were statistically evaluated using a Student’s *t*-test. Significance was at *P*-values of < 0.05.

## Results

### NCKAP1 Expression is Significantly Decreased in ccRCC and Correlated With Patient Outcomes

**FIGURE 1:**
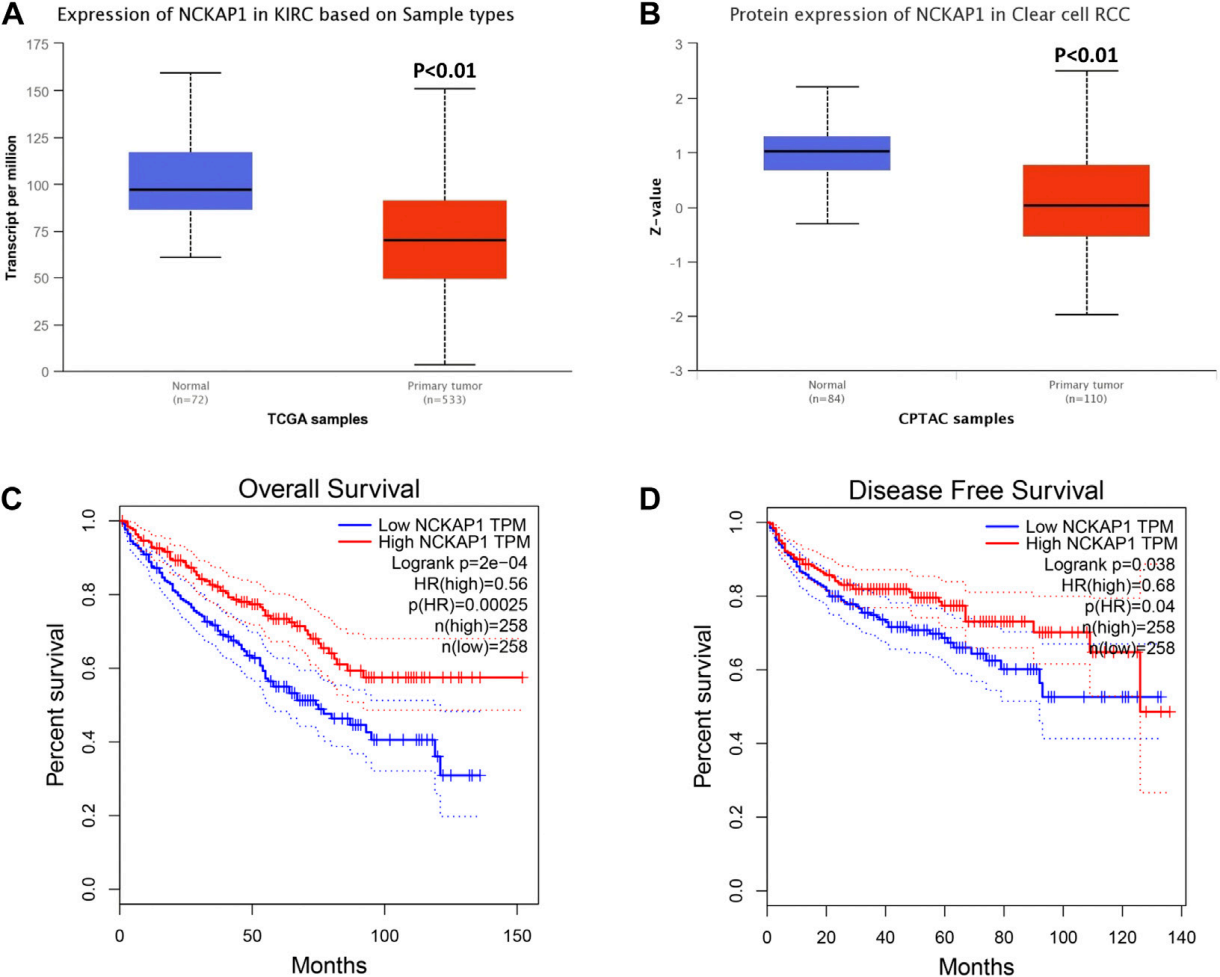
Analysis of NCKAP1 expression and survival curve in ccRCC on the basis of the UALCAN database and GEPIA survival analysis platform. **(A)** mRNA expression and **(B)** protein expression of NCKAP1 in ccRCC and normal tissues. The overall survival **(C)** and disease-free survival **(D)** curve of NCKAP1 in ccRCC. *p < 0.05 is statistically significant.

Using the UALCAN database, we evaluated NCKAP1 mRNA and protein expression in ccRCC. We found that NCKAP1 mRNA expression was decreased in ccRCC tissues compared to normal tissues, consistent with NCKAP1 protein expression data (Figures 1A,B). To investigate the prognostic value of NCKAP1, we used the GEPIA database to determine NCKAP1 OS and DFS between mRNA expression and ccRCC were analyzed. Figures 1C,D show that low expression of NCKAP1 mRNA may indicate worse OS and DFS in ccRCC.

### Correlation of NCKAP1 Expression With Clinical Features of ccRCC

**FIGURE 2:**
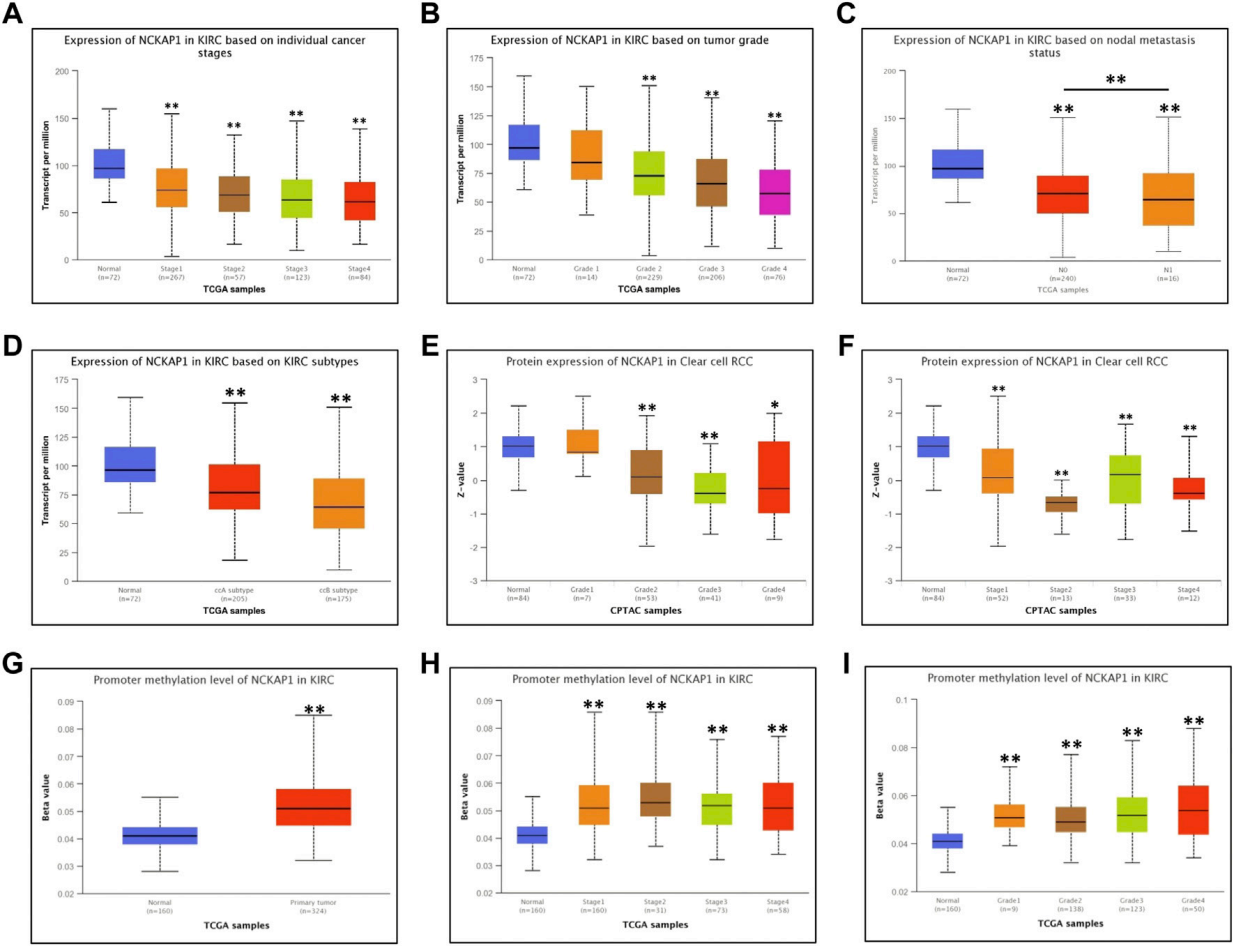
Correlation between NCKAP1 expression and clinicopathologic characteristics in ccRCC tissues. mRNA expression of NCKAP1 in ccRCC sub-groups based on individual cancer stages **(A)**, tumor grade **(B)**, node metastasis **(C)** and ccRCC subtypes **(D)**; protein expression of NCKAP1 in ccRCC sub-groups based on individual cancer stages **(E)** and tumor grade **(F)**. Promoter methylation level of NCKAP1 in normal tissues and ccRCC **(G)**; Promoter methylation level of NCKAP1 in ccRCC of individual tumor stage and tumor grade **(H,I)**. *p < 0.05 and **p < 0.001 are statistically significant.

Using the UALCAN database, we examined the correlation between NCKAP1 expression and various clinicopathological features of ccRCC and found that NCKAP1 mRNA expression was significantly associated with tumor grade, TNM stage, and lymph node metastasis (Figures 2A–C). These data were similar to the results observed for NCKAP1 protein expression (Figures 2E,F); NCKAP1 mRNA expression was lower in advanced cancers compared to early-stage cancers (ccA subtype vs. ccB subtype; *p* < 0.001). Furthermore, methylation levels of the NCKAP1 promoter were increased in ccRCC compared to normal tissue (Figure 2G), confirming that this was the opposite of NCKAP1 expression. ccRCC stages, tumor grades 1, 2, 3, and 4 also showed elevated NCKAP1 promoter methylation levels (Figures 2H,I).

**FIGURE 3:**
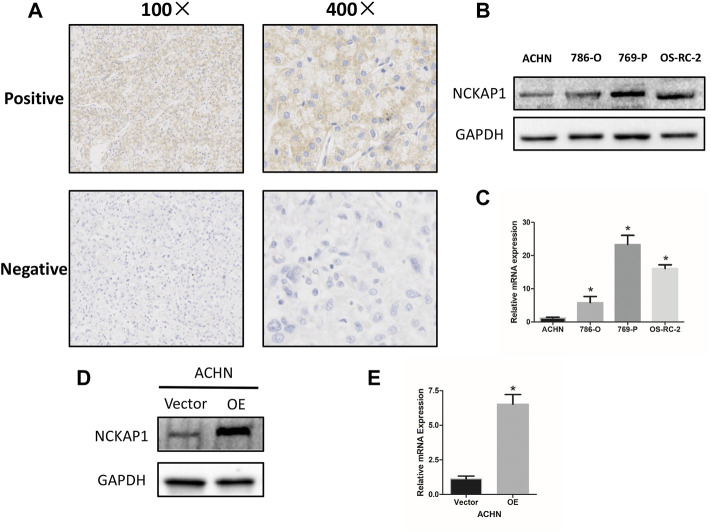
NCKAP1 expression in ccRCC tissues and cell lines. **(A)** Positive (upper) and negative (lower) expressions of NCKAP1in ccRCC. (×100 left, ×400 right). **(B)**Western blotting and **(C)** Quantitative real-time PCR (qPCR) results show that ACHN cells exhibited low expression compared to that of 786-O, 769-P and OS-RC-2 cells. GAPDH was used as a loading control. Overexpression of NCKAP1 (OE) in a transfected ACHN cell line verified by western blotting **(D)** and qPCR **(E)** compared to that of ACHN cells transfected with the control vector (Vector). GAPDH was used as a loading control.

To confirm the association between NCAKP1 expression and the clinical features in ccRCC, IHC staining of tissue microarrays was performed. Division of the 75 specimens on the TMA was done into a negative NCKAP1 expression group (*n* = 53) and a positive NCKAP1 expression group (*n* = 22) (Figure 3A). The correlations between NCKAP1 expression and clinical features are summarized in Table 1. A significant association was observed between low NCKAP1 expression and TNM stage, tumor size and pathological grade, whereas NCKAP1 expression displayed no apparent associations with age, gender and tumor position.

**TABLE 1:** Relationship between NCKAP1 expression and clinicopathological factors in ccRCC.

Characteristics	Number	Negative	Positive	P Value
*n*	75	53	22	
Age				0.144
≦55	28	17	11	
⟩55	47	36	11	
Gender				0.525
Male	47	32	15	
Female	28	21	7	
Position				0.776
Left	36	26	10	
Right	39	27	12	
TNM Stage				0.039
I + II	52	33	19	
III + IV	23	20	3	
Tumor Size				0.315
≦7 cm	41	27	14	
⟩7 cm	34	26	8	
Grade				0.027
I + II	55	35	20	
III + IV	20	18	2	

### NCKAP1 Expression in ccRCC Cell Lines and Overexpression of NCKAP1 in ACHN Cells

A significant association was found between low expression of NCKAP1 and clinicopathological features, suggesting that NCKAP1 may be very much involved in ccRCC tumorigenesis. We examined the expression of NCKAP1 in the ccRCC cell lines described above and found that ACHN cells had significantly lower levels of NCKAP1 expression at the mRNA and protein levels relative to other cell lines (Figures 3B,C). Next, for further functional verification, an overexpression plasmid (pEZ-Lv201-NCKAP1) or control vector (pEZLv201) was transfected into ACHN cells. After transfection, qRT-PCR and Western blotting were employed to confirm the expression levels of NCKAP1 mRNA and protein, respectively (Figures 3D,E).

### Overexpression of NCKAP1 Targets the Ability of ACHN Cells to Proliferate, Migrate, and Invade

**FIGURE 4:**
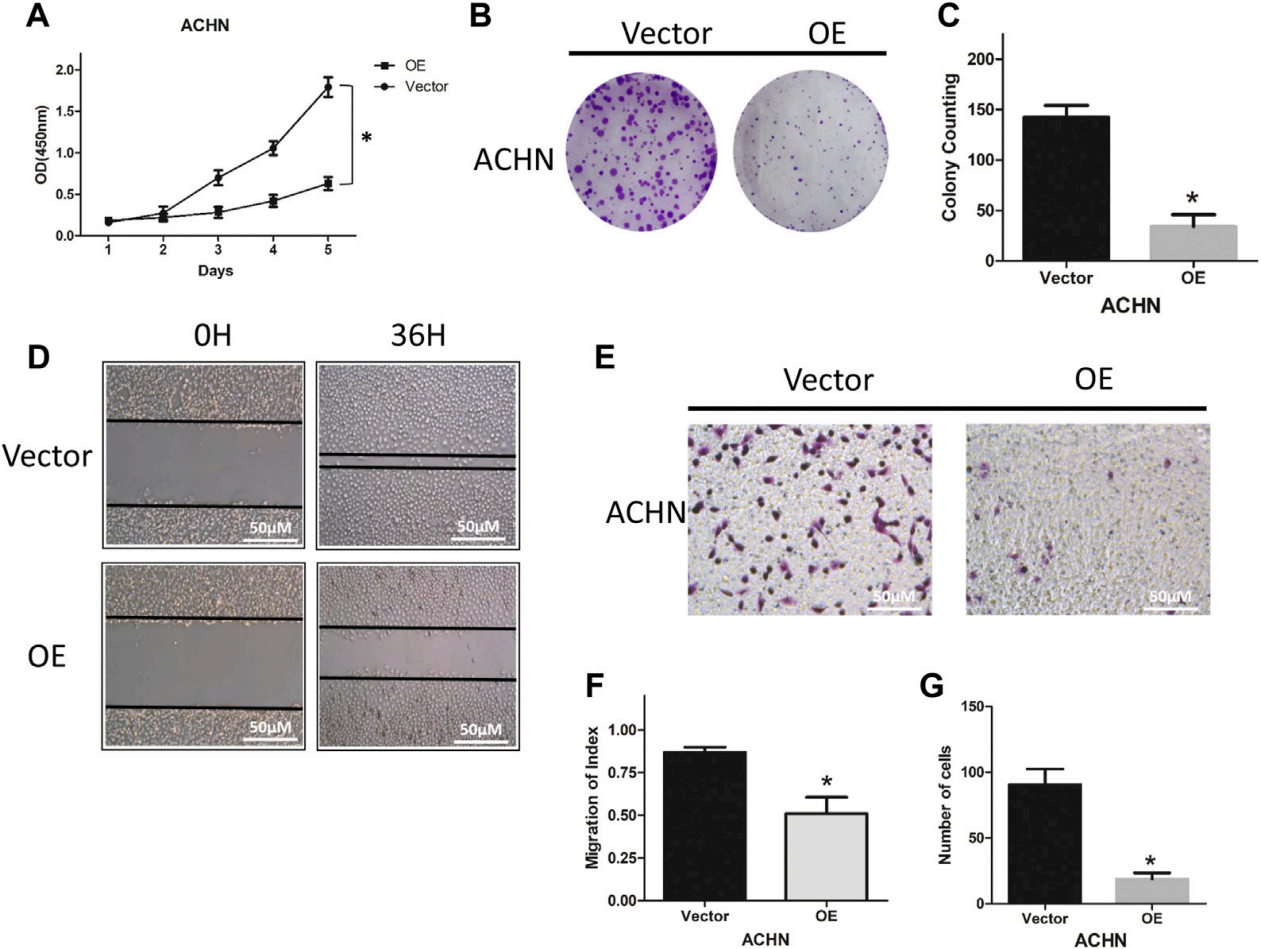
NCKAP1 inhibited cell growth, migration and invasion *in vitro*. CCK-8 assay results showed the cell viability in **(A)** ACHN-OE cells compared with ACHN-Vector cells. Cell colony formation assay showed a statistically significant decrease of **(B,C)** ACHN-OE cells compared with ACHN-Vector cells. Wound-healing assay results showed a statistically significant decrease of migration **(D,F)** ACHN-OE cells compared with ACHN-Vector cells. Scale bar = 50 μm. Transwell invasion assay results showed a statistically significant decrease of invaded **(E,G)** ACHN-OE cells compared with ACHN-Vector cells. Scale bar = 50 μm. The results are mean ± SD values from three independent experiments, *p < 0.05.

To explore the functional role of NCKAP1, we characterized the ability of ACHN cells overexpressing NCKAP1 to proliferate, migrate, and invade. As shown in Figures 4A–C, overexpression of NCKAP1 in ACHN cells suppressed the cell growth rate and reduced the number and size of colonies formed. The number and size of colonies formed were reduced compared to the vector control group. Overexpression of NCKAP1 also reduced wound closure (Figures 4D,F) and invasiveness (Figures 4E,G) relative to vector controls. These data indicate a potentially significant effect of NCKAP1 on the ability of ACHN cells to proliferate, migrate, and invade.

### Overexpression NCKAP1 inhibits RCC Progression *in vivo*

**FIGURE 5:**
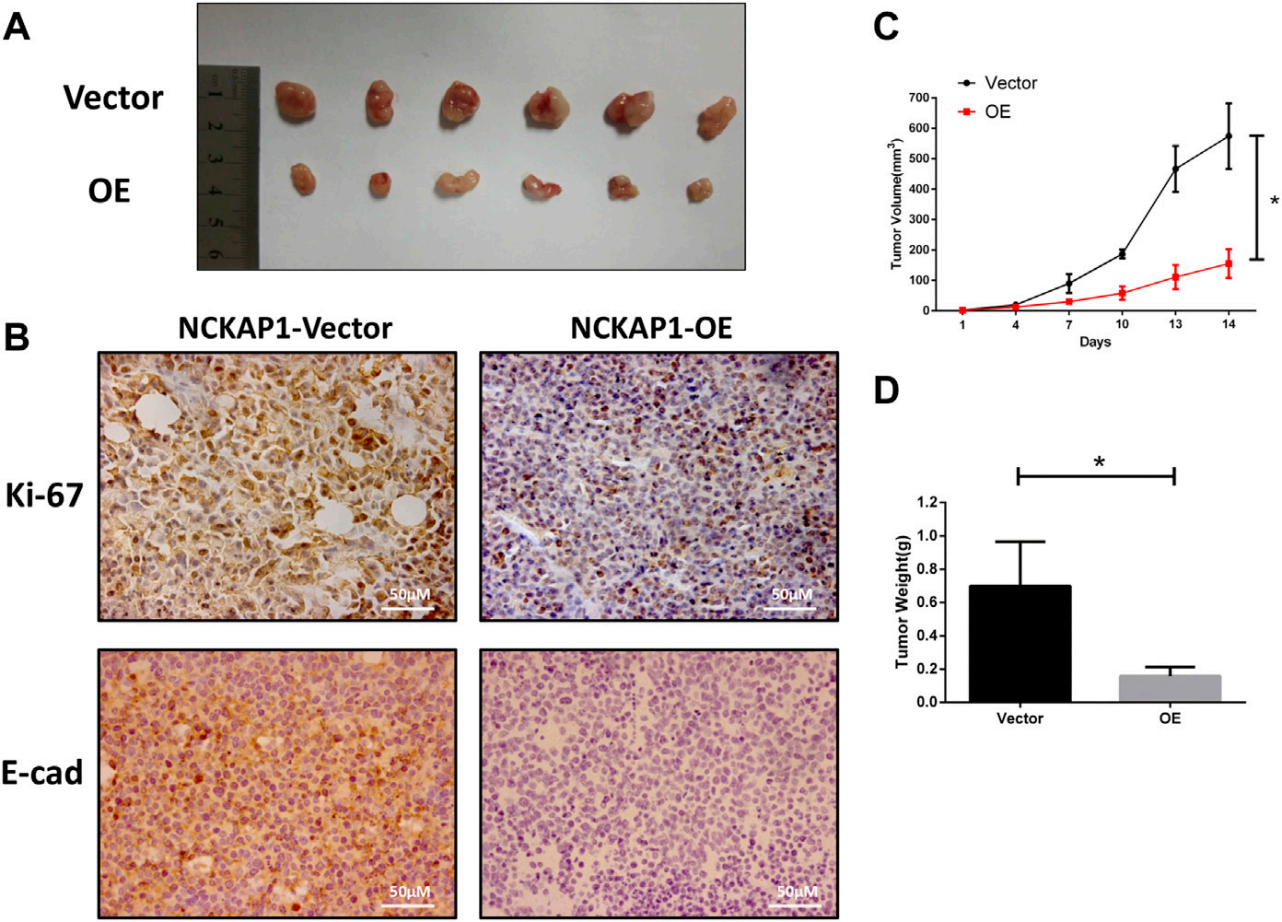
NCKAP1 suppressed RCC progression *in vivo*. **(A)** Representative Data showed that NCKAP1-OE significantly inhabited tomor growth in nude mice xenograft. **(B)** Immunohistochemistry (IHC) staining showed that the expression of Ki-67 and E-CAD differed in tumor tissues. (Scale bar = 50 μm) **(C)** Tumor volume and **(D)** tumor weight was decreased significantly in NCKAP1-OE cells’ mice model, *p < 0.05.

We investigated the impact of NCKAP1 overexpression on the tumor growth properties of ACHN cells by establishing a xenograft model established in nude mice. We found that overexpression of NCKAP1 inhibited the formation of tumors compared to vector cells (Figure 5A). The growth rate and weights in the ACHN-OE mice were distinctly lower against the control group (Figures 5B,C, *p* < 0.05) indicating that NCKAP1 can suppress the growth of RCC tumor xenografts. Morever, in contrast with the NCKAP1-Vector, the NCKAP1-OE group significantly decreased expression of Ki-67 and E-cad in tumor tissues (Figure 5B). Taken together, these results demonstrated that NCKAP1 inhibited the growth and metastasis of ACHN tumor.

### Enrichment Analysis of NCKAP1-Related Co-expressed Genes in ccRCC

**FIGURE 6:**
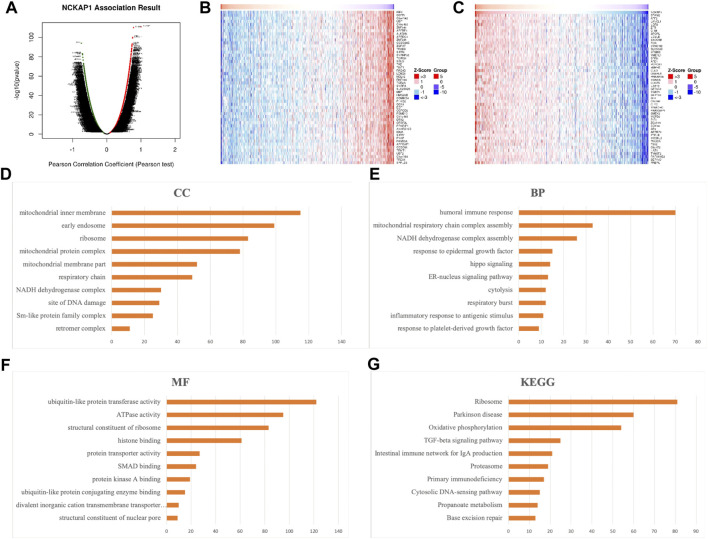
Significant genes correlated with NCKAP1 and enrichment analysis in RCC (LinkedOmics). **(A)** The positive and negative genes correlated with NCKAP1 in ccRCC (Pearson test). **(B)** The top 50 positive genes and **(C)** the top negative genes correlated with NCKAP1 in ccRCC. Red indicated positively correlated genes, while green indicated negatively correlated genes. **(D)** Cellular components. **(E)** Biological processes. **(F)** Molecular functions. **(G)** KEGG pathway analysis.

To explore potential molecular mechanisms of NCKAP1 in ccRCC, we investigated NCKAP1-related coexpressed genes and determined their enrichment function in LinkedOmics using data from 533 TCGA ccRCC patients. Volcano plots showing these up- and down-regulated genes are shown in Figure 6A; the top 50 genes positively and negatively associated with NCKAP1 are shown as heat maps in Figures 6B,C. As shown in Supplementary Figure S1, STAM2 (Cor = 0.7816, *p* = 6.515e-111), ATF2 (Cor = 0.7585, *p* = 8.011e-101) and LANCL1 (Cor = 0.7568, *p* = 3.944e-100) showed strong positive correlation with NCKAP1; IRF3 (Cor = −0.7433, *p* = 8.929e-95), OGFR (Cor = −0.7132, *p* = 5.77e-84) and C9orf142 (Cor = −0.7126, *p* = 8.602e-84) showed strong negative correlation with NCKAP1

### GO Enrichment Analysis Was Performed to Explore the Cellular Components, Biological Processes, and Molecular Functions of NCKAP1

The results (Figures 6D–F) revealed that genes significantly correlated with NCKAP1 are primarily involved in the liquid immune response, ribosomal NADGH dehydrogenase, mitochondrial respiratory chain complex assembly, and ATPase activity. KEGG pathway analysis (Figure 6G) revealed that NCKAP1 is involved in the enriched in ribosomal signaling, oxidative phosphorylation, TGF-β signaling pathway, cytoplasmic DNA-sensing pathway, and IgA synthesis immune network in the intestine. In addition, NCKAP1 and the top three significant positive or negative genes, including STAM2, ATF2, LANCL1, IRF3, OGFR, and C9orf142, were selected as hub genes for pathway analysis by the GSCALite platform. We explored the role of hub genes in well-known cancer-related pathways including TSC/mTOR, RTK, RAS/MAPK, PI3K/AKT, hormone ER, hormone AR, EMT, DNA damage response, cell cycle, and apoptosis pathway. We found that NCAKP1 is involved in the activation of PI3K/AKT, RTK, Hormone ER, RAS/MAPK pathway, and Hormone AR, DNA Damage Response, Cell Cycle pathway, whereas NCAKP1 is involved in the inhibition of EMT and TSC/mTOR pathways. Furthermore, by using GEPIA2 database, NCKAP1 expression was found to be significantly positively related to the expression of EMT signaling genes VIM (*p* = 3.9e-07), E-cad (*p* = 3.2e-05), and N-cad (*p* = 0) in the TCGA-KIRC cohort (Supplementary Figure S4). These results are shown in Supplementary Figure S2, S4.

### Regulatory Factor NCKAP1 Network in ccRCC

The NCKAP1 network of kinase targets in ccRCC was examined. As shown in Supplementary Table S1, the top five kinases are mainly Ataxia Telangiectasia Mutated (ATM), Ribosomal Protein S6 Kinase B1 (RPS6KB1), cyclin-dependent kinase 1 (CDK1), cyclin-dependent kinase 5 (CDK5) and Large Tumor Suppressor Kinase 1 (LATS1). We also explored potential miRNA targets of NCKAP1 in ccRCC (Supplementary Table S1). The top five miRNA targets were identified as (ATGTTAA) MIR-302C, (CTTGTAT) MIR-381, (ATAGGAA) MIR-202, (GTATTAT) MIR-369–3P and (ATATGCA) MIR-448.

### Characterization of Immune Cells and NCKAP1 Expression in CcRCC

**FIGURE 7:**
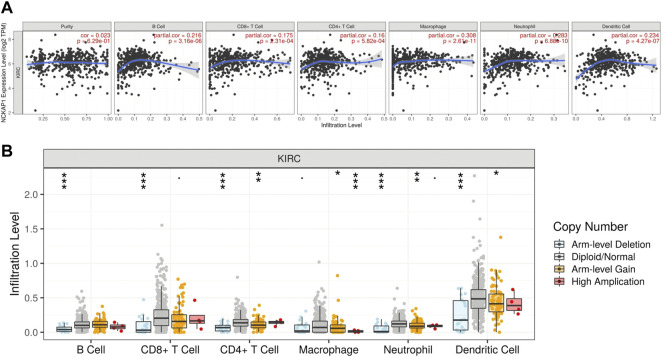
Correlation between NCKAP1and immune cell infiltration in ccRCC. **(A)** The correlation between NCKAP1 and the immune infiltration level in ccRCC (TIMER). **(B)** The correlation between copy number alteration of NCKAP1 and immune cell infiltration in ccRCC.

Tumor immunity is extremely involved in tumorigenesis and prognosis in ccRCC Using the TIMER web portal, we correlated NCKAP1 expression with the intensity of immune infiltrating cells NCKAP1 expression was found in B cells (Cor = 0. 216, *p* = 3.16e-06), CD8^+^ T cells (Cor = 0. 175, *p* = 2.31e-04), macrophages (Cor = 0.308, *p* = 2.61e-11), neutrophils (Cor = 0.283, *p* = 6.88e-10) and dendritic cells (Cor = 0.234, *p* = 4.27e-07), showing a clear positive correlation (Figure 7A). Furthermore, somatic copy number changes of NCKAP1 can indeed inhibit the infiltration of immune cells such as CD8^+^ T cells, B cells, neutrophils, dendritic cells, macrophages and CD4^+^ T cells in ccRCC (Figure 7B). After adjusting by tumor purity, we found an important biomarker of immune cell correction by NCKAP1. As shown in Table 2, markers of Monocyte (CD86, CSF1R), TAM (IL10), M1 macrophage (NOS2, PTGS2), M2 macrophage (CD163, VSIG4, MS4A4A) and Dendritic cell (NRP1) which showed significant correlations with NCKAP1 were obtained. Similar results were obtained for Th1 (STAT1), Th2 (STAT6), Tfh (BCL6), Th17 (STAT3), and Treg (CCR8, STAT5B). In summary, we expected a specific correlation between NCKAP1 expression and the intensity of immune infiltration.

**TABLE 2:** Correlation analysis between NCAKP1 and gene biomarkers of immune cells in ccRCC (TIMER).

Immune Cells	Biomarkers	None		Purity	
		Cor	*p*-Value	Cor	*p*-Value
CD8+T cell	CD8A	−0.096	2.62e-02	0.009	8.50e-1
	CD8B	−0.195	*	−0.067	1.52e-01
T cell (general)	CD3D	−0.234	**	−0.104	2.60e-02
	CD3E	−0.252	***	−0.126	7.22e-03
	CD2	−0.159	*	−0.002	9.72e-01
B cell	CD19	−0.159	*	−0.056	2.32e-01
	CD79A	−0.235	**	−0.134	3.98e-03
Monocyte	CD86	0.013	7.82e-01	0.196	*
	CD115(CSF1R)	−0.004	9.27e-01	0.156	*
TAM	CCL2	−0.063	1.74e-01	0.074	1.12e-01
	CD68	0.02	6.57e-01	0.125	7.64e-03
	IL10	0.07	1.32e-01	0.218	**
M1 macrophage	INOS(NOS2)	0.004	9.37e-01	0.01	8.29e-01
	IRF5	−0.104	2.47e-02	0.033	4.81e-01
	COX2(PTGS2)	0.201	**	0.251	***
M2 macrophage	CD163	0.147	1.44e-03	0.299	***
	VSIG4	0.036	4.34e-01	0.162	*
	MS4A4A	0.057	2.14e-01	0.213	**
Neutrophils	CD66b(CEACAM8)	0.113	1.45e-02	0.112	1.61e-02
	CD11b(ITGAM)	0.003	9.41e-01	0.128	6.20e-03
	CCR7	−0.177	*	−0.029	5.38e-01
NK	KIR2DL1	−0.109	1.79e-02	−0.037	4.25e-01
	KIR2DL3	−0.128	5.24e-03	−0.027	5.64e-01
	KIR2DL4	−0.17	*	−0.067	1.54e-01
	KIR3DL1	−0.149	1.21e-03	−0.065	1.68e-01
	KIR3DL2	−0.199	**	−0.086	6.48e-02
	KIR3DL3	−0.108	1.93e-02	−0.077	1.02e-01
	KIR2DS4	−0.161	4.4e-04	−0.098	3.60e-02
Dendritic cell	HLA−DPB1	−0.174	*	−0.029	5.41e-01
	HLA−DQB1	−0.167	2.8e-04	−0.031	5.02e-01
	HLA−DRA	−0.105	2.33e-02	0.061	1.31e-01
	HLA−DPA1	−0.14	2.29e-03	−0.003	9.41e-01
	BDCA−1(CD1C)	−0.075	1.02e-01	0.062	1.88e-01
	BDCA−4(NRP1)	0.313	***	0.4	***
	CD11c(ITGAX)	−0.084	6.91e-02	0.04	3.90e-01
Th1	T−bet (TBX21)	−0.16	4.86e-04	−0.016	7.27e-01
	STAT4	0.018	6.92e-01	0.176	*
	STAT1	0.267	***	0.399	***
	IFN−γ(IFNG)	−0.047	3.04e-01	0.104	2.59e-02
	TNF−α(TNF)	−0.098	3.31e-02	0.046	3.27e-01
Th2	GATA3	−0.137	2.84e-03	0.035	4.50e-01
	STAT6	0.249	***	0.25	***
	STAT5A	0.076	9.76e-02	0.086	6.69e-02
	IL13	−0.056	2.25e-01	−0.005	9.13e-01
Tfh	BCL6	0.168	*	0.239	**
	IL21	0.03	5.21e-01	0.125	7.25e-03
Th17	STAT3	0.389	***	0.438	***
	IL17A	−0.033	4.8e-01	−0.008	8.65e-01
Treg	FOXP3	−0.211	**	−0.08	8.68e-02
	CCR8	0.092	4.53e-02	0.264	***
	STAT5B	0.298	***	0.31	***
	TGFβ(TGFB1)	−0.034	4.57e-01	0.059	2.09e-01
T cell exhaustion	PD−1(PDCD1)	−0.228	**	−0.11	1.84e-02
	CTLA4	−0.073	1.14e-01	0.042	3.67e-01
	LAG3	−0.203	*	−0.085	7.03e-02
	TIM3(HAVCR2)	−0.029	5.34e-01	0.15	1.30e-03
	GZMB	−0.214	**	−0.086	6.50e-02

*p < 0.05. **p < 0.01. ***p < 0.001

### Immune Checkpoint, TMB, MSI, and NCKAP1 Expression in ccRCC

**FIGURE 8:**
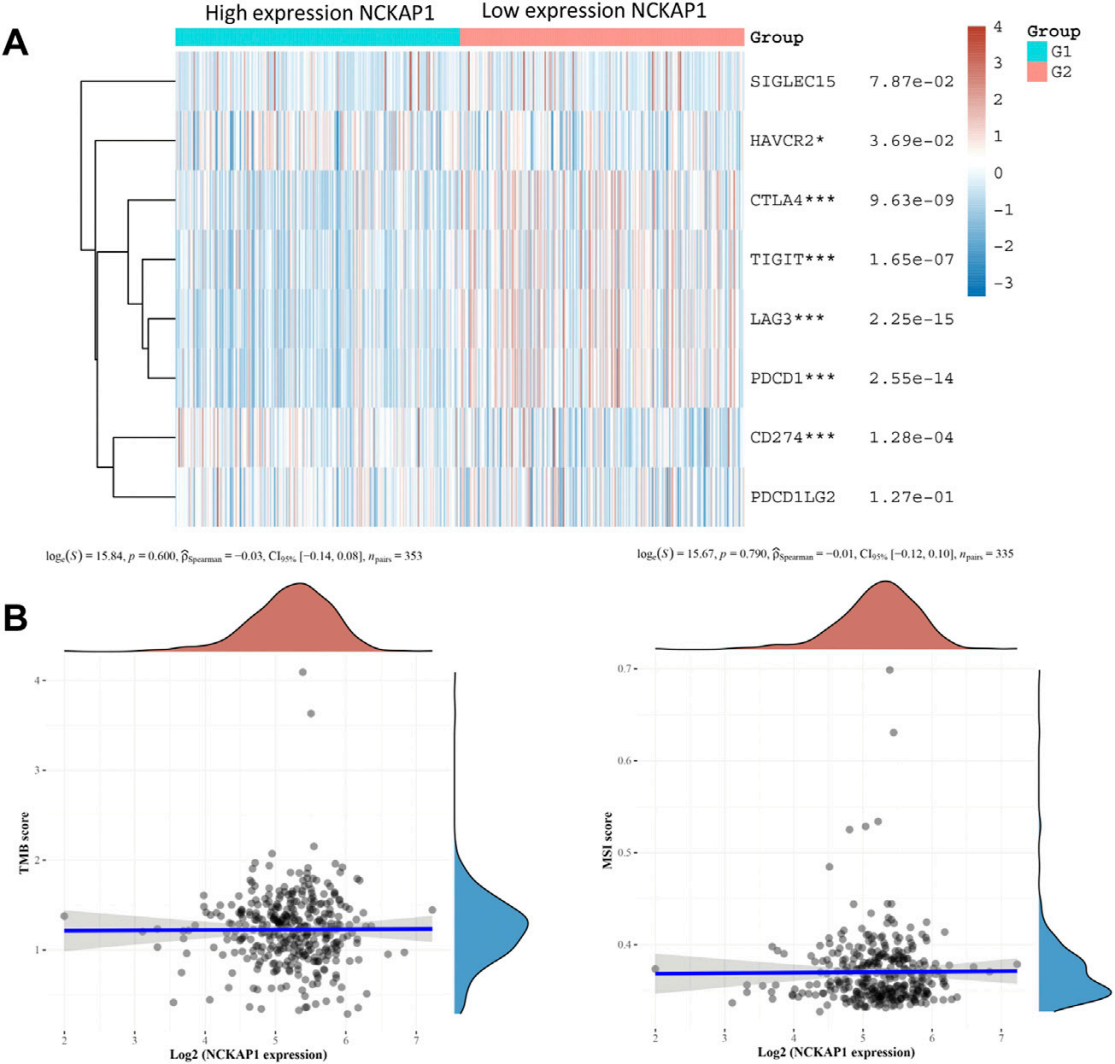
Association of the NCKAP1 with immune checkpoints, TMB, and MSI in ccRCC. **(A)** Correlation of NCKAP1 expression with immune checkpoint genes. **(B)** Correlation of NCKAP1 expression with tumor mutational burden (TMB). Correlation of NCKAP1 expression with microsatellite instability (MSI).

Immune checkpoints play an essential role in targeted immunotherapy and are considered an important method of tumor therapy. In this study, we analyzed the expression of NCKAP1 and SIGLEC15, HAVCR2, CTLA4, TIGIT, PDCD1LG2, CD274, LAG3, and PDCD1 immune checkpoint-related genes. The results showed that immune checkpoint markers (HAVCR2, CTLA4, TIGIT, CD274, LAG3, PDCD1) were significantly correlated with NCKAP1 expression; TMB and MSI were considered prognostic markers to predict response to immunotherapy in CCRCC (Pang et al., 2016). However, our results showed that TMB and MSI were not significantly correlated with NCKAP1 (Figure 8B).

## Discussion

ccRCC is one of the universal urinary tract malignancies (Chen et al., 2016; Pang et al., 2016). ccRCC is challenging to diagnose early, and patients often present with advanced metastatic cancer (Rydzanicz et al., 2013). Therefore, identifying new biomarkers in ccRCC that can be used as a therapeutic system for early diagnosis and new treatment design is much needed.

Previous studies have shown that NCKAP1 is associated with multiple cancer types; high NCKAP1 levels are clearly associated with the clinical features of human non-small cell lung cancer (NSCLC) (Zhu et al., 2021). NCKAP1 also interacts with HSP90 (heat shock protein 90) and is highly implicated in NSCLC cell invasion and metastasis (Xiong et al., 2019a). John et al. used a mouse xenograft breast cancer metastasis model to show that NCKAP1 plays a critical role in invasion and metastasis by regulating WASF3 stability and function (Cowell et al., 2017), and Karthic et al. found that targeted deletion of NCKAP1 inhibited melanoma progression using a BRAF/PTEN-deficient mouse model (Swaminathan et al., 2021). Zhu et al. also analyzed gene expression profiles in the GEO database and showed that NCKAP1 is an autophagy-related gene and is significantly associated with event-free survival in several melanoma patients (Zhu et al., 2019). Xiao et al. found that the miR-34c-3p target NCKAP1 promotes the progression of hepatocellular carcinoma and is associated with poor prognosis (Xiao et al., 2017). These results indicate that NCKAP1 may function as an oncogene in a variety of cancer types. On the other hand, NCKAP1 has been shown to have tumor suppressor activity that regulates the HCC cell cycle through the regulation of Rb1/p53, but not the WASF pathway (Zhong et al., 2019). These differences may be due to selective activation of target genes of NCKAP1-related pathways in specific tissues.

The association between NCKAP1 expression and clinical features in ccRCC patients remains largely unknown. We first detected NCKAP1 mRNA and protein expression levels using the TCGA and CPTAC datasets and found that NCKAP1 expression was downregulated in tumors compared to normal tissues. When compared between NCKAP1 expression, tumor grade, TNM stage, and lymph node metastasis, a negative correlation was observed, suggesting that NCKAP1 may have an antitumor effect in ccRCC. Other studies have shown that transcriptional repression of tumor suppressor genes occurs with tumor progression due to hypermethylation of promoter regions (Ruiz de la Cruz et al., 2021). Therefore, we speculated that low expression of NCAKP1 might be associated with high methylation levels in ccRCC, which is consistent with our previous results. Our IHC results showed that low expression of NCKAP1 correlated with tumor size, stage, and grade, confirming our previous findings. GEPIA survival analysis showed that low NCKAP1 expression was significantly associated with poor prognosis in ccRCC patients. Functional assays also showed that NCKAP1 affects cancer cell proliferation, migration, and invasion, as well as inhibits tumor growth *in vivo*.

Next, we examined the expression of genes significantly associated with NCKAP1 and its function in ccRCC. The results showed that genes associated with NCKAP1 function primarily in humoral immune responses, NADH dehydrogenase and mitochondrial respiratory chain complex assembly, oxidative phosphorylation, cytoplasmic DNA sensing and TGF-β signaling pathways. It has been shown that among these biological processes, immune response and metabolic alterations played essential functions in tumorigenesis (Smith et al., 2018; Monette et al., 2019; Chakraborty et al., 2021). The TGF-β signaling pathway is frequently downregulated in tumor cells and may increase or hinder tumor growth (Bao et al., 2021). Prior studies have shown that TGF-β can modify tumor activity by inhibiting host tumor immune surveillance and directly regulating oncogenic metabolism in EMT, cellular invasion and metastasis (Huber-Ruano et al., 2017; Lee et al., 2017; Ungefroren, 2019). Epithelialmesenchymal transition (EMT), which play vital roles intumor cell migration and invasion, is essential step in the process of metastasis. To our best knowedge, WAVE complex has been shown to be a promoter of cell invasion in various cancer cell types. NCKAP1, as a part of WAVE complex, it is therefore required for WAVE function and its regulation of invasion. Targeting NCKAP1 is thus reported to lead to the suppression of metastasis (Teng et al., 2016). Taken together, NCKAP1 and its related genes are primarily involved in tumor-related functions and playing important roles in EMT-related signaling pathways, suggesting that NCKAP1 may mediate ccRCC tumorigenesis and development.

Tumor-infiltrating immune cells are a crucial element in the tumor microenvironment and have been found to influence proliferation, invasion, and metastasis in various cancer types (Bremnes et al., 2016; Xiong et al., 2019b; Yang et al., 2020). Tumor-infiltrating immune cells and immune checkpoints are thought to play an essential role in immunotherapy, making them hotspot studies in ccRCC treatment (Xu et al., 2021; Wu et al., 2022). Our results revealed a clear correlation between NCKAP1 levels and immune cell counts and biomarker levels. Furthermore, the association with various immune checkpoints (HAVCR2, CTLA4, TIGIT, CD274, LAG3, and PDCD1) strongly suggested that NCKAP1 is a co-regulator of immune checkpoints in ccRCC. These results suggest that NCKAP1 may play an essential role in regulating tumor immune invasion and immunotherapy, which in turn may affect the prognosis of ccRCC.

This study has several limitations, and future studies should validate these results in a more significant number of cases and thoroughly investigate the detailed mechanisms by which NCKAP1 is involved in ccRCC.

## Conclusion

In this study, using multiple portal databases, we found that low NCKAP1 expression levels were negatively correlated with clinical features and prognosis of ccRCC. Furthermore, our results indicate that NCAKP1 may play an important role in oncogenesis *in vitro* and *in vivo*. In addition, NCKAP1 and its related genes function primarily in metabolic-related signaling pathways and immune cell infiltration, predictably suggesting that NCKAP1 is a prognostic biomarker for ccRCC.

## Data Availability

The original contributions presented in the study are included in the article/Supplementary Material, further inquiries can be directed to the corresponding authors.
