# Supplementary material for: NCKAP1 is a Prognostic Biomarker for Inhibition of Cell Growth in Clear Cell Renal Cell Carcinoma
Source: Front Genet. 2022 Jul 26;13:764957. doi: 10.3389/fgene.2022.764957 (PMC9360572; doi:10.3389/fgene.2022.764957)
Supplement: Supplementary file 1 [file Table1.DOCX]

**Supplement Table 1. The Kinase, and miRNA-target networks of NCKAP1 in KIRC (LinkedOmics).**

| \| **Enriched Category** \| \| --- \| | **Geneset** | **LeadingEdgeNum** | **P Value** |
| --- | --- | --- | --- | --- |
| **Kinase Target** | Kinase_ ATM  Kinase_RPS6KB1  Kinase_CDK1  Kinase_CDK5  Kinase_LATS1 | 123  23  258  66  5 | 0  0  0  0.01  0.004 |
| **miRNA Target** | ATGTTAA, MIR-302C  CTTGTAT, MIR-381  ATAGGAA, MIR-202  GTATTAT, MIR-369-3P  ATATGCA, MIR-448 | 99  91  58  101  83 | 0  0  0  0  0 |
