# Supplementary figures and images for: NCKAP1 is a Prognostic Biomarker for Inhibition of Cell Growth in Clear Cell Renal Cell Carcinoma
Source: Front Genet. 2022 Jul 26;13:764957. doi: 10.3389/fgene.2022.764957 (PMC9360572; doi:10.3389/fgene.2022.764957)

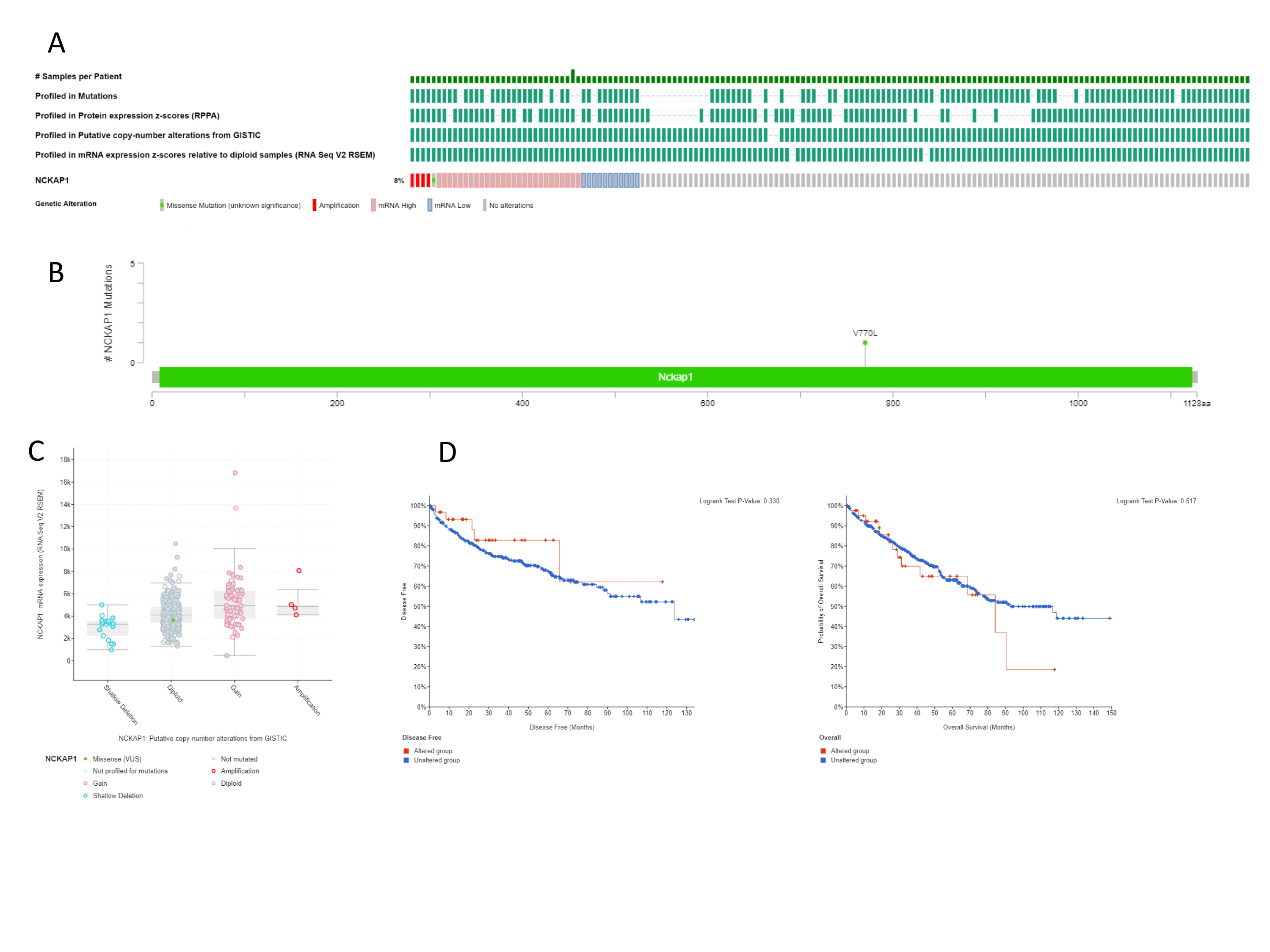

Supplement: Supplementary file 2 [file Image3.TIF]

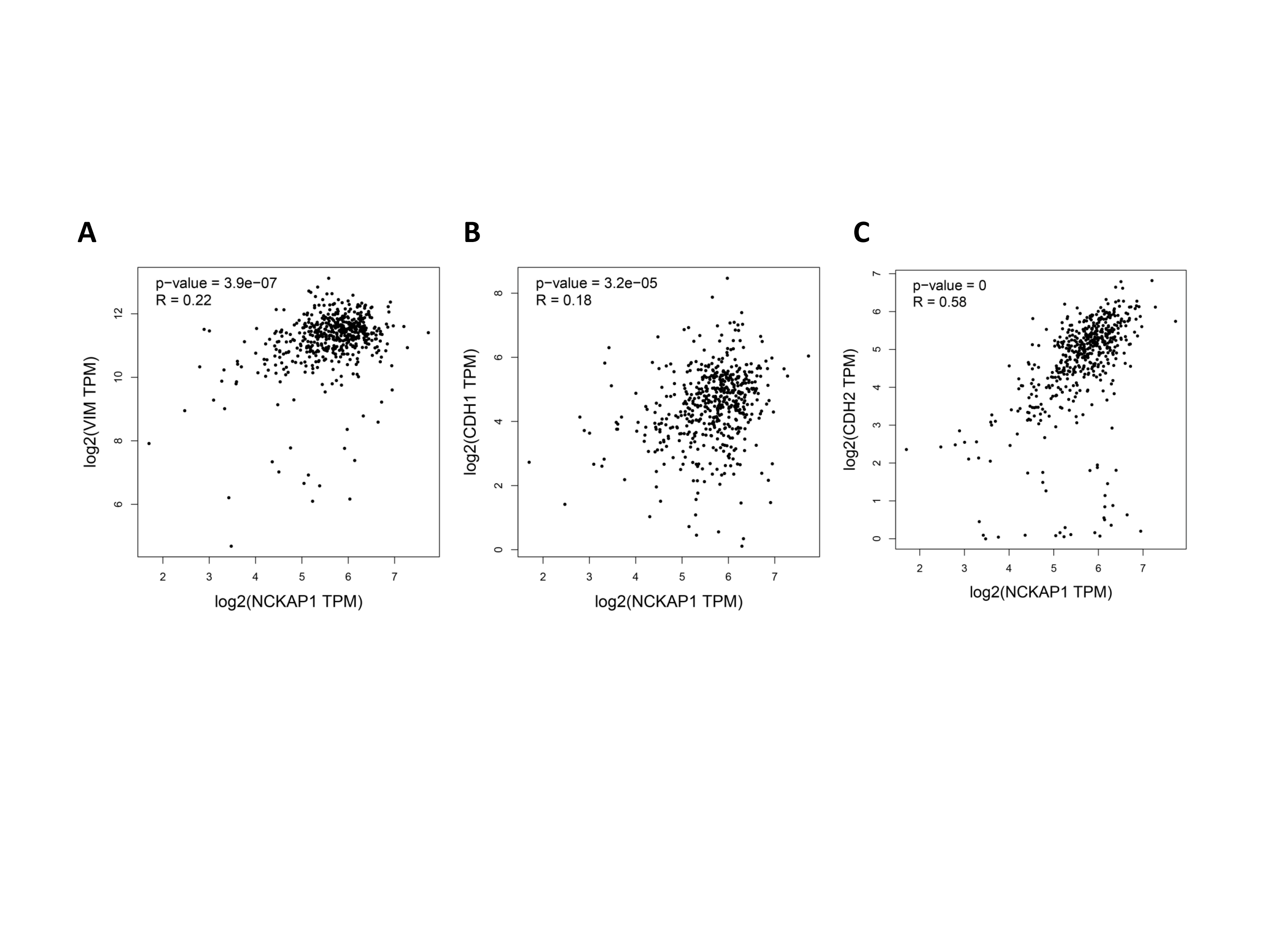

Supplement: Supplementary file 3 [file Image4.TIF]

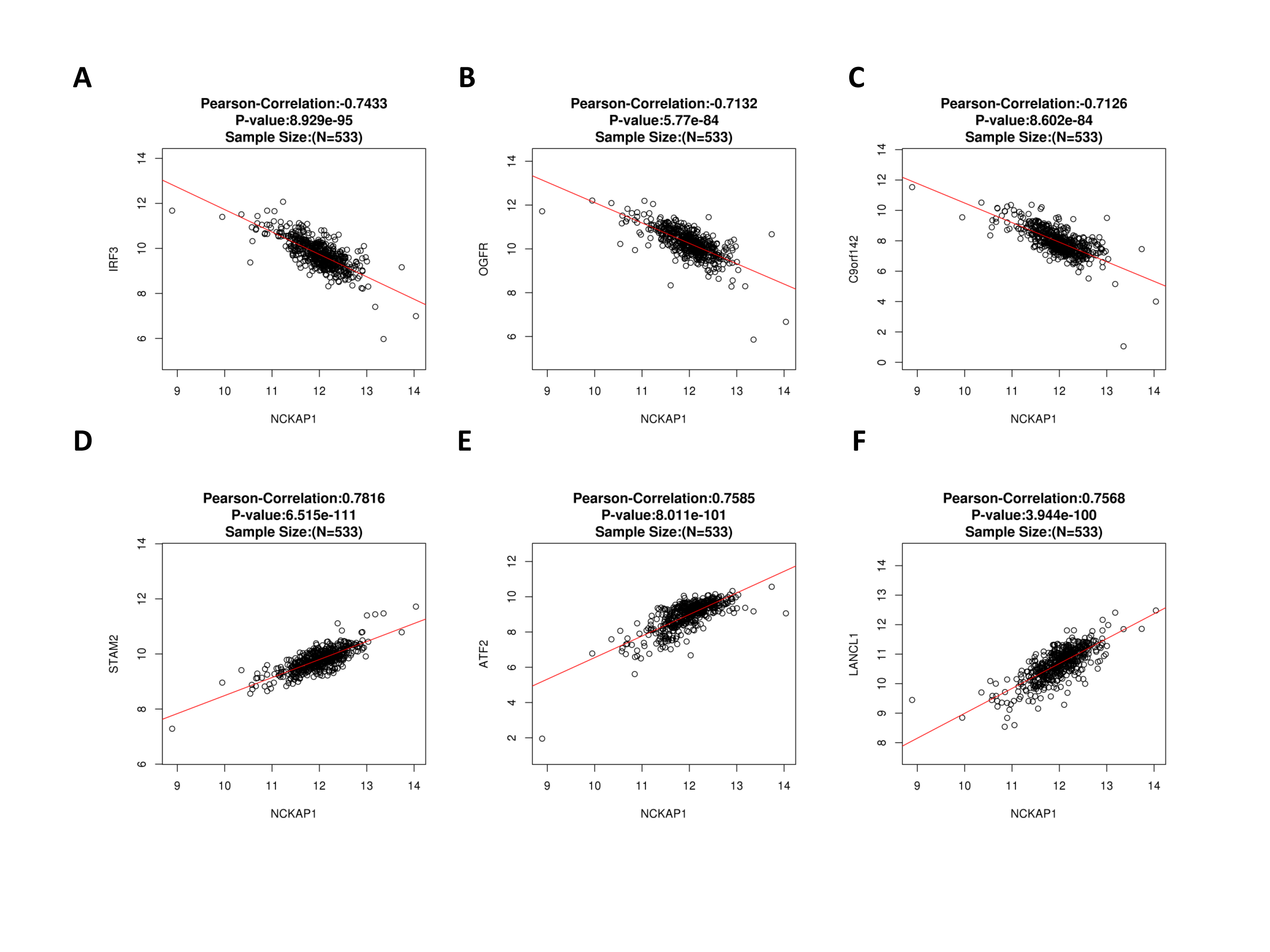

Supplement: Supplementary file 4 [file Image1.TIF]

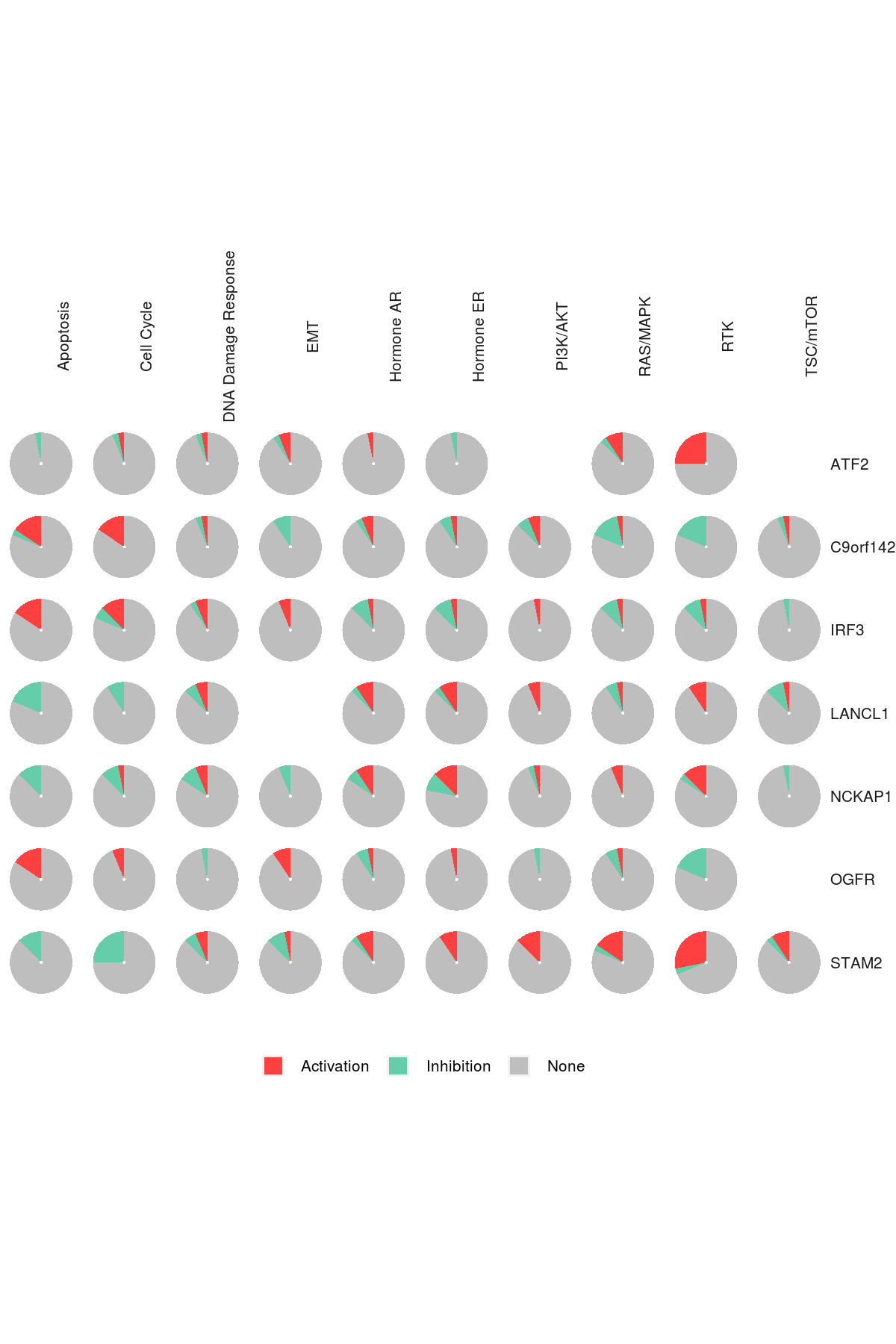

Supplement: Supplementary file 5 [file Image2.PNG]
